# Supplementary material for: Heterologous Systemic Prime–Intranasal Boosting Using a Spore SARS-CoV-2 Vaccine Confers Mucosal Immunity and Cross-Reactive Antibodies in Mice as well as Protection in Hamsters
Source: Vaccines (Basel). 2022 Nov 10;10(11):1900. doi: 10.3390/vaccines10111900 (PMC9692796; doi:10.3390/vaccines10111900)
Supplement: Supplementary file 1 [file vaccines-10-01900-s001.zip › vaccines-1993144-supplementary.pdf]

A

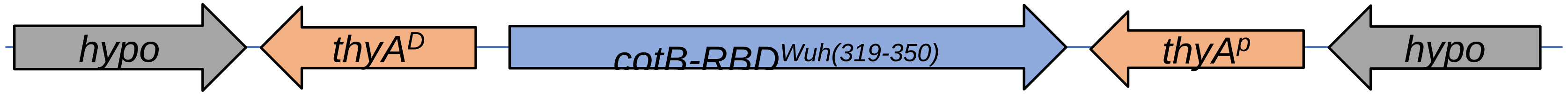

CotB-RBD$^{Wuh(319-530)}$ MW = 61.8 kDa.

MSKRRMKYHSNNEISYYNFLHSMKDKIVTVYRGGPESKKGKLTAVKSDYIALQAEKKIIYYQLEHVKSITEDTNNSTTTIETEEMLDADDFHSLIGHLINQSVQFNQGGPESKKGRLVWLGDD
YAALNTNEDGVVYFNIHHIKSISKHEPDLKIEEQTPVGVLEADDLSEVFKSLTHKWVSINRGGPEAIEGILVDNADGHYTIVKNQEVLRIYPFHIKSISLGPKGSYKKEDQKNEQNQEDNNDKD
SNSFISSKSYSSSKSSKRSLKSSDDQSSKSGRSSRSKSSSKSSKRSLKSSDYQSSKSGRSSRSKSSSKSSKRSLKSSDYQSSKSSKRSPRSRVQPTESIVRFPNITNLCPFGEVFNATRFA
SVYAWNRKRISNCVADYSVLYNSASFSTFKCYGVSPTKLNDLCFTNVYADSFVIRGDEVRQIAPGQTGKIADYNYKLPDDFTGCVIAWNSNNLDSKVGGNYNYLYRLFRKSNLKPFERDIST
EIYQAGSTPCNGVEGFNCYFPLQSYGFQPTNGVGYQPYRVVVLSFELLHAPATVCGPKKS

B

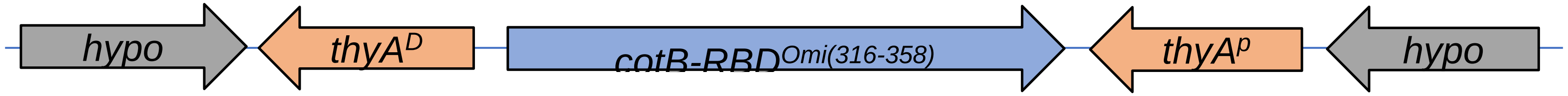

CotB-RBD$^{Omi(316-538)}$ MW = 63.36 kDa.

MSKRRMKYHSNNEISYYNFLHSMKDKIVTVYRGGPESKKGKLTAVKSDYIALQAEKKIIYYQLEHVKSITEDTNNSTTTIETEEMLDADDFHSLIGHLINQSVQFNQGGPESKKGRLVWLGDD
YAALNTNEDGVVYFNIHHIKSISKHEPDLKIEEQTPVGVLEADDLSEVFKSLTHKWVSINRGGPEAIEGILVDNADGHYTIVKNQEVLRIYPFHIKSISLGPKGSYKKEDQKNEQNQEDNNDKD
SNSFISSKSYSSSKSSKRSLKSSDDQSSKSGRSSRSKSSSKSSKRSLKSSDYQSSKSGRSSRSKSSSKSSKRSLKSSDYQSSKSSKRSPRSRVQPTESIVRFPNITNLCPFDEVFNATRFA
SVYAWNRKRISNCVADYSVLYNLAPFFTFKCYGVSPTKLNDLCFTNVYADSFVIRGDEVRQIAPGQTGNIADYNYKLPDDFTGCVIAWNSNKLDSKVSGNYNYLYRLFRKSNLKPFERDIST
EIYQAGNKPCNGVAGFNCYFPLRSYSFRPTYGVGHQPYRVVVLSFELLHAPATVCGPKKSTNLVKNKCVNF

C

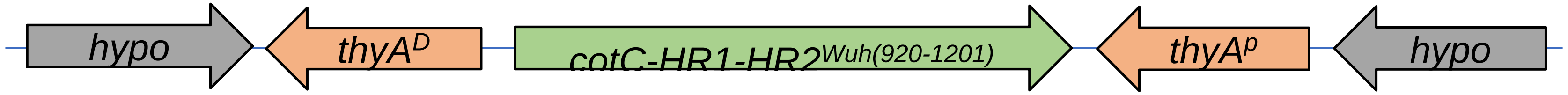

CotC-HR1-HR2$^{Wuh(920-1201)}$ MW = 39.87 kDa.

MGYYKKYKEEYYTVKKTYYKKYYEYDKKDYDCDYDKKYDDYDKKYYDHDKKDYDYVVEYKKHKKHYQKLIANQFNSAIGKIQDSLSSTASALGKLQDVVNQNAQALNTLVKQLSSNFGAIS
SVLNDILSRLDKVEAEVQIDRLITGRLQSLQTYVTQQLIRAAEIRASANLAATKMSECVLGQSKRVDFCGKGYHLMSFPQSAPHGVVFLHVTYVPAQEKNFTTAPAICHDGKAHFPREGVFV
SNGTHWFVTQRNFYEPQIITTDNTFVSGNCDVVIGIVNNTVYDPLQPELDSFKEELDKYFKNHTSPDVDLGDISGINASVVNIQKEIDRLNEVAKNLNESLIDLQ

Supplementary Figure S1: Recombinant Spore Vaccines

Chimeric genes inserted at the *thyA* loci of *B. subtilis* are shown. The Wuhan and Omicron-derived Spike RBD domains were fused in frame to the C-termini of the CotB spore coat protein and the HR1-HR2 domain (Wuhan) of Spike fused in frame to the C-terminus of the CotC spore coat protein. The 15 amino acid changes present in the Omicron RBD variant are highlighted. The Mwof the chimera are shown.

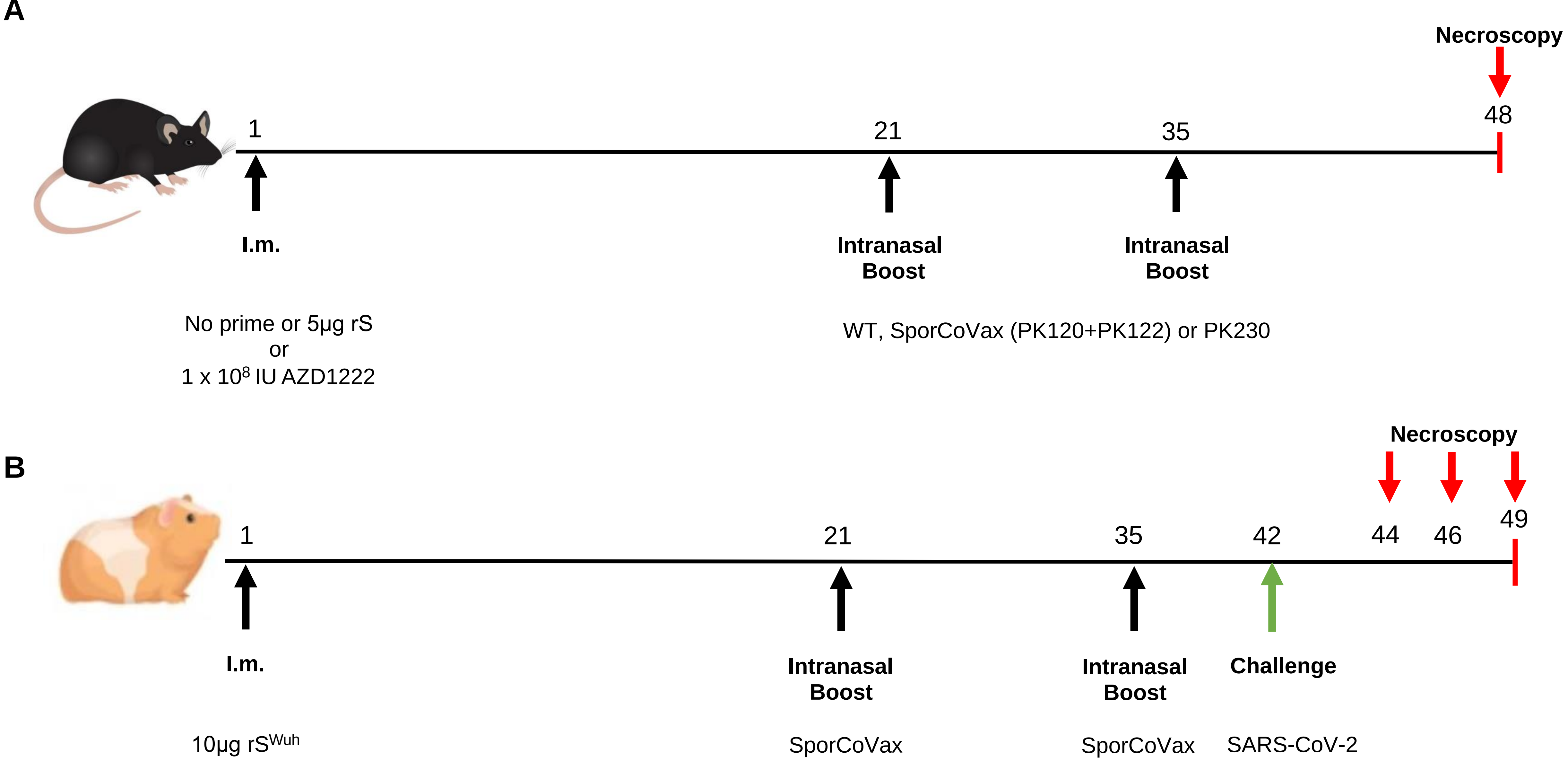

Supplementary Figure S2: Animal Study Schedules

For murine immunisations intra-nasal boosts were given three and five weeks after the prime. Each boost consisted of three daily administrations of 10 μL/mouse, 5 μL/nare). Boost 1, days 21, 22, 23 and Boost 2, days 35, 36, 37). For hamster boosts animals were given a single intranasal dose of 0.1 mL (50 mL/nare) three and five weeks post-prime.

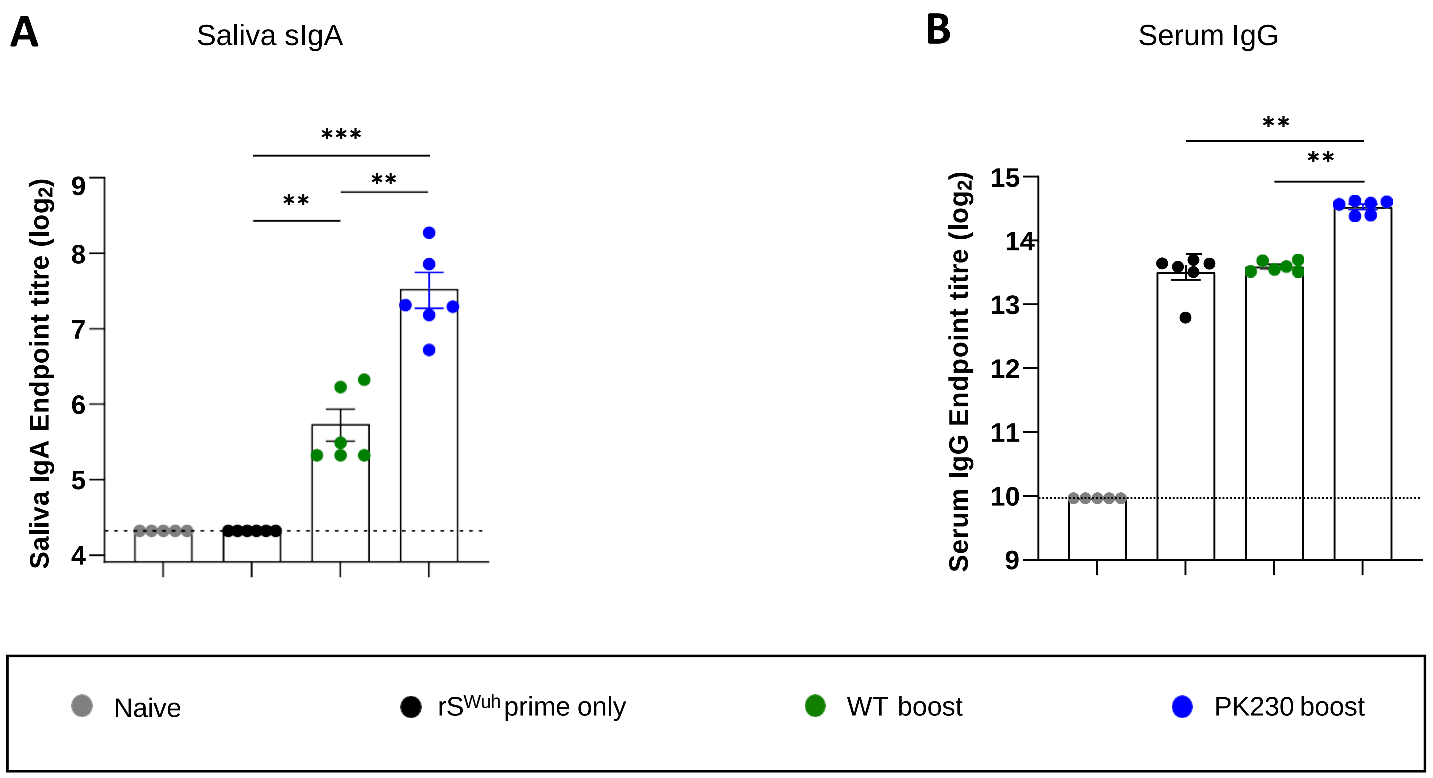

Supplementary Figure S3: Intranasal Boost of the AZD1222 Vaccine with Spores Expressing Omicron-specific Antigens

Three groups (n=6) of mice (female Balb/c) were primed (i.m.) with the AZD1222 vaccine. Two groups were then boosted (intranasal) with spores (5×10^9 CFU/dose) spores of either WT (naked spores, no antigen expression) or PK230 (CotB-RBD^{Omi}) at 21 and 35-days post-prime. Panels show rS^{Omi}-specific responses determined by ELISA (OD_{450nm}) 48-days post-immunization. ***Panel A***) rS^{Omi}-specific sIgA in longitudinal saliva samples, ***B***) rS^{Omi}-specific IgG in serum. Significance was tested using a two-tailed Mann-Whitney U test, **p < 0.01, *** p < 0.001
